# Development and validation of an extended Cox prognostic model for patients with ER/PR+ and HER2− breast cancer: a retrospective cohort study

**DOI:** 10.1186/s12957-022-02790-0

**Published:** 2022-10-12

**Authors:** Yiqun Xie, Xizhou Li, Ying Wu, Wenting Cui, Yang Liu

**Affiliations:** 1 Department of Breast Surgery, Huangpu Branch of Shanghai Ninth People’s Hospital, Shanghai Jiao Tong University School of Medicine, Shanghai, 200011 China; 2 Department of General Surgery, Changhai Hospital, Naval Medical University (Second Military Medical University), Shanghai, 200433 China; 3 School of Statistics, East China Normal University, Shanghai, 200062 China

**Keywords:** Breast cancer, Estrogen receptor, Progesterone receptor, Human epidermal growth factor receptor 2, Clinicopathological prognostic factor, Prognostic model

## Abstract

**Background:**

The purpose of this study was to explore a new estrogen receptor (ER) and/or progesterone receptor (PR)+ and human epidermal growth factor receptor 2 (HER2)− breast cancer prognostic model, called the extended Cox prognostic model, for determining the cutoff values for multiple continuous prognostic factors and their interaction via the new model concept and variable selection method.

**Methods:**

A total of 335 patients with ER/PR+ and HER2− breast cancer were enrolled for the final analysis. The primary endpoint was breast cancer-specific mortality (BCSM). Prognostic factors (histological grade, histological type, stage, T, N, lymphovascular invasion (LVI), P53, Ki67, ER, PR, and age) were included in this study. The four continuous variables (Ki67, ER, PR, and age) were partitioned into a series of binary variables that were fitted in the multivariate Cox analysis. A smoothly clipped absolute deviation (SCAD) variable selection method was used. Model performance was expressed in discrimination and calibration.

**Results:**

We developed an extended Cox model with a time threshold of 164-week (more than 3 years) postoperation and developed a user-friendly nomogram based on our extended Cox model to facilitate clinical application. We found that the cutoff values for PR, Ki67, and age were 20%, 60%, and 41–55 years, respectively. There was an interaction between age and PR for patients aged ≥ 41 years and *PR* ≥ 20% at 164-week postoperation: the older the patients with ER/PR+, HER2−, and *PR* ≥ 20% were, the lower the survival and more likely to recur and metastasize exceeding 164 weeks (more than 3 years) after surgery.

**Conclusions:**

Our study offers guidance on the prognosis of patients with ER/PR+ and HER2− breast cancer in China. The new concept can inform modeling and the determination of cutoff values of prognostic factors in the future.

**Supplementary Information:**

The online version contains supplementary material available at 10.1186/s12957-022-02790-0.

## Background

Breast cancer is the malignant carcinoma with the highest incidence among Chinese women. In China, with an increase in breast cancer in recent years, research on prognostic models for breast cancer has become a growing concern [1]. Recently, prognostic gene signatures (Oncotype DX, MammaPrint, and so on) have been taken more seriously; however, the current prognostic gene signatures are not ready to be used in clinical practice due to a plethora of concerns regarding cost and technology, regardless of first- or second-generation gene signatures [2–4]. In the USA, Oncotype DX is the most commonly used breast cancer genomic assay and costs around US $4000. It is used in only approximately one-third of patients with breast cancer in America and in less than 20% of patients in European countries [5]. In China, a 21-gene assay without a standardized test norm is commonly used and costs around US $500–$1000 (not covered by Chinese healthcare insurance). The test is performed in approximately 20 to 100% of patients with breast cancer at different hospitals in China; the instances where 100% of breast cancer patients were tested were mainly clinical trials. Furthermore, recently, the prognostic value of the classic clinicopathologic variables is being reconsidered [3–8]. Some evidence indicates that clinicopathologic variable models are excellent surrogates for prognostic gene signatures [3, 5]. Hence, classic clinicopathologic variable models are highly valued due to their feasibility for clinical practice. From 2006 to the present, the National Comprehensive Cancer Network has classified invasive breast cancer into four subtypes [9]. The molecular subtype of breast cancer is a classification method similar to the intrinsic subtype and is more suitable for current clinical practice in China, also serving as an independent prognostic factor [9–11]. Among the four molecular subtypes of breast cancer, estrogen receptor (ER) and/or progesterone receptor (PR) + and human epidermal growth factor receptor 2 (HER2)− occurred most commonly and account for approximately 60% of breast cancer patients [12]. There are urgent demands and wider potential impacts for exploration of improved prognostic models for patients with ER/PR+ and HER2− breast cancers based on classic clinicopathologic variables, particularly in China.

The current classic prognostic algorithms (PREDICT, Adjuvant! Online, and Nottingham Prognostic Index) are far from ideal [13–15]. These models were often based on datasets from non-Chinese or non-Asian patients. Specifically, it was assumed that these prognostic factors, including Ki67 (a nuclear marker of cell proliferation), ER, PR, and age, were continuous factors, or it was assumed that the cutoff values of prognostic factors, including Ki67, ER, PR, and age, were determined merely based on univariate analysis, experience, or speculation. In addition, existing models ignored the interaction effects between prognostic factors. Therefore, current models showed poor accuracy and were not suitable for clinical practice in China. It is critical to develop a novel-improved prognostic algorithm to analyze clinical data from China.

For this reason, in this study, we selected 335 patients with ER/PR+ and HER2− breast cancer to explore our new ER/PR+ and HER2− breast cancer prognostic model using classical clinicopathologic variables, called the extended Cox prognostic model. The cutoff values for multiple continuous prognostic factors were determined, and the interaction effect between the factors was elucidated via the new modeling concept and variable selection method.

### Patients and methods

#### Study population

**Fig. 1:**
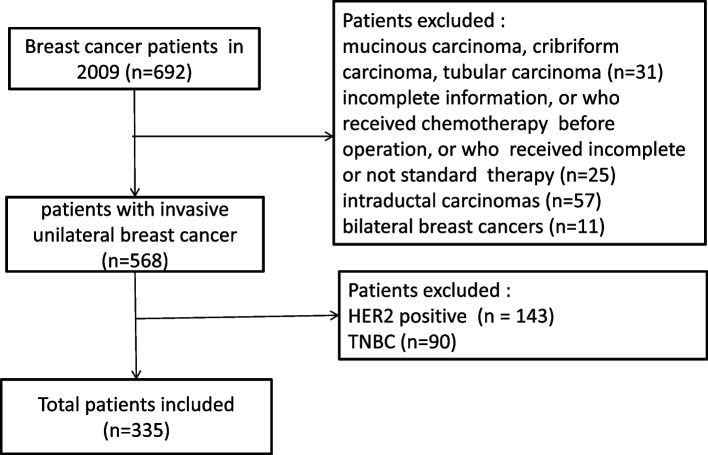
Patient’s selection

All patients with invasive unilateral breast cancer admitted to the Department of Breast Surgery at Huangpu Branch, Shanghai Ninth People’s Hospital, Shanghai Jiao Tong University School of Medicine from January 2009 to December 2009 were evaluated. Information available from medical records included age at diagnosis, number of lymph nodes sampled, and number of positive lymph nodes (categorized as 0, 1 to 3, 4 to 9, and 10+ nodes positive [6]), lymphovascular invasion (LVI) (categorized as positive or negative), tumor size (categorized as < 21 mm, 21 to 50 mm, 50+ mm [6]), histological grade (categorized as I, II, III [6]), pathological type, protein 53 (p53) status (categorized as positive or negative), proliferating cell nuclear antigen Ki67 (Ki67) status, ER status, PR status, HER2 status, information on local therapy (wide local excision, mastectomy, radiotherapy), and type of adjuvant systemic therapy (chemotherapy, endocrine therapy, or both). Among the total 692 patients, those with any one of the following conditions were excluded from the analyses: mucinous carcinoma, cribriform carcinoma, or tubular carcinoma [9], those with incomplete information, who received chemotherapy or radiation before operation, who did not undergo surgery, who did not complete local treatment (local excision without radiotherapy), who had no axillary lymph node dissection, who did not complete adjuvant systemic therapy (chemotherapy and endocrine therapy), and intraductal carcinomas, or bilateral breast cancers, leaving 568 individuals. Afterward, the ER status, PR status, and HER2 status were detected. Among the 568 patients, 143 patients with HER2+ breast cancer and 90 patients with triple-negative breast cancer (TNBC) were excluded from this study. Finally, the 335 individuals among the 568 patients were determined to be ER/PR+ and HER2− type, comprising our study population. Variables for each patient included age, TNM status, T stage, N stage, pathological subtype, histology grade, LVI, p53 status, Ki67 status, ER status, PR status, vital status, and survival time (Fig. 1).

### Immunohistochemistry and fluorescence in situ hybridization

We performed the preparation of tissue section again and reassessed ER status, PR status, HER2 status, p53 status, and Ki67 status for all the samples using immunohistochemistry (IHC) and fluorescence in situ hybridization (FISH). All formalin-fixed paraffin-embedded tumor blocks were collected at the time of surgery prior to adjuvant therapy and were stored at room temperature. Approval was obtained from the Institutional Review Boards. Tumor sections of 4 to 5 μm were cut. One section was stained with hematoxylin/eosin to confirm the presence of invasive carcinoma, and the other sections were used for IHC and FISH by two independent pathologists.

IHC: Immunohistochemistry was performed using a Ventana BenchMark Ultra autostainer according to the manufacturer’s instructions. Rabbit monoclonal primary antibodies to ER (clone: SP1), PR (clone: 1E2), HER2 (clone: 4B5), and Ki67 (clone: 30-9) were purchased from Roche. Rabbit monoclonal primary antibodies to p53 (clone: D0-7) were purchased from Gene. UltraView Universal DAB detection kit (catalogue no. 760-500, Ventana Medical System, Arizona, USA) was used.

FISH: Formalin-fixed paraffin-embedded sections were dewaxed and rehydrated. Then, the sections were heated at 100 °C for 20 min in a water bath and digested with proteinase K for 10 min. HER2/CEP17 probes (LBP, 2101001) were denatured on slides at 85 °C and hybridized at 37 °C overnight. Nuclei were counterstained with DAPI and then observed under a fluorescence microscope.

### Categorization of patients into ER/PR+ and HER2− subtypes of breast cancer

Subtypes were assessed according to the ER/PR immunohistochemistry and HER2 immunohistochemistry or FISH status. The criteria for evaluating ER and PR in breast cancer cells by IHC [16] were as follows: ER or PR was positive if the cell nuclei showed a brown color. In one section, five high-power regions were selected randomly. The patient was assigned to subtype ER/PR+ if the percentage of positive cells was ≥ 1% in these regions. The criteria for evaluating HER2 by IHC were as follows: the patients were categorized into four subtypes: 0, 1+, 2+, or 3+ [17]. Subtypes HER2 IHC 0 and 1+ were HER2 negative (HER2−). Subtype HER2 IHC 2+ was equivocal in HER2 status. Finally, subtype HER2 IHC 3+ was HER2 positive. Tumor cells of HER2 IHC 2+ subtype were further analyzed by fluorescence in situ hybridization (FISH). According to the HER2/chromosome 17 centromere ratio and the average HER2 gene copy number, FISH results were considered positive, equivocal, or negative [17]. Patients with equivocal FISH results were excluded from the study. Among the 568 patients, the HER2 status of 185 individuals was detected as HER2 IHC (++) [17]. Tissue microarrays (TMAs) were constructed from the tissue core with a diameter of 1.5 mm to detect the HER2 status of these 185 patients using FISH methods. Finally, the 335 individuals among the 568 patients were determined to be ER/PR+ and HER2− type, comprising our study population (Fig. 1).

### p53 status and Ki67 status

The criteria for evaluating p53 status in breast cancer cells by IHC were as follows: p53 was positive if the cell nuclei showed a brown color. In one section, five high-power regions were selected randomly. The patient was assigned to subtype p53+ if the percentage of positive cells was ≥ 10% in these regions. The criteria for evaluating Ki67 status in breast cancer cells by IHC were as follows: Ki67 was positive if the cell nuclei showed a brown color. Ki67 status in cancerous tissue areas was quantified as 1–100, which was the percentage of tumor nuclei positive (positive nuclei) over all the tumor nuclei (positive nuclei and negative nuclei) by manual counting.

### Treatment for the patients

All patients underwent modified radical mastectomy/breast-conserving surgery and adjuvant chemotherapy. They were treated with six cycles of CEF (cyclophosphamide, epirubicin, and fluorouracil) chemotherapy, four cycles of CEF followed by four cycles of T (docetaxel) chemotherapy, or four to six cycles of TEC (docetaxel, epirubicin, and cyclophosphamide) chemotherapy after surgery. If necessary, patients received postoperative radiotherapy followed by endocrine therapy, but not trastuzumab treatment. Endocrine therapy had been performed in accordance with the contemporary domestic guideline at our hospital from 2009.

### Follow-up

We defined breast cancer-specific mortality (BCSM) as mortality from breast cancer [18]. The primary endpoint of this study was BCSM, which was determined by following up the survival of patients over a certain time period. From the first day after surgery until death or the end of the study (September 15, 2016), we made telephone calls and/or outpatient visits every 3 months to follow-up on the survival status of the patients and the cause of death. The follow-up records were double-checked with those from the Department of Cancer Control and Prevention, Shanghai Municipal Center for Disease Control and Prevention.

### Statistical analysis

The extended Cox prognostic model was developed in all eligible patients as follows. First, to determine the cutoff values for each continuous variable (Ki67, ER, PR, and age), these four continuous variables were partitioned into a series of binary variables. Second, all variables were fitted in the multivariate Cox analysis. A smooth clipped absolute deviation (SCAD) variable selection method [19] was used to build a Cox prognostic model to determine the independent variables, cutoff values, and interaction effect between different factors. Finally, when developing the model, we hypothesized that the model could be divided into two parts by a certain time point. We built a new model, named the extended Cox prognostic model, with a time threshold of 164 weeks (more than 3 years) based on the smallest Akaike information criterion (AIC) value.

We evaluated the predictive accuracy of the extended Cox prognostic model based on the parameters of discrimination and calibration. For model discrimination, receiver operating characteristic (ROC) curves were plotted for the data at 1-year, 3-year, and 5-year postoperation [20]. We also calculated the areas under the ROC curves (AUCs). Model calibration was assessed by a simplified goodness-of-fit (GOF) method [21]. We compared the number of deaths observed and calculated at 5-year postoperation. We grouped the risk scores into 10 sets and then calculated the GOF statistics for each set [22]. This provided a GOF chi-square test. All analyses were conducted using R software version 3.3.2.

## Results

### Patient characteristics

All 335 patients were females. All clinical and pathological data of the patients were complete, including age, tumor size, axillary lymph node status, LVI status, histological grade, pathological type, ER/PR/HER2/Ki67/P53 status, and surgical and postoperative adjuvant treatment (specified as chemotherapy, targeted therapy, radiotherapy, and endocrine therapy). Patient characteristics and differences in characteristics by survival outcome are detailed in Table 1. Among 335 patients, 22, 267, and 46 had histologic grades 1, 2, and 3 tumors, respectively [6]. According to the 2017 American Joint Committee on Cancer staging system [6], 206, 126, 3, and 0 patients had stages T1, T2, T3, and T4 disease, respectively, while 180, 98, 41, and 16 patients had stages N0, N1, N2, and N3 disease, respectively.

**Table 1:** Characteristics of the included patients

Characteristic	Full cohort (*n* = 335)	%	Censored (*n* = 307)	Died (*n* = 28)
Age				
Median age (range)	53 (26–89)		53 (26–89)	56 (34–80)
< 41	27	8.1	23	4
41–55	159	47.5	153	6
≥ 55	149	44.5	131	18
Histologic grade				
I	22	6.6	22	0
II	267	79.7	251	16
III	46	13.7	34	12
Pathological type				
Invasive carcinoma of no special type	270	80.6	247	23
Invasive lobular carcinoma	15	4.5	14	1
Mixed carcinoma	14	4.2	14	0
Micropapillary carcinoma	15	4.5	12	3
Invasive papillary carcinoma	5	1.5	4	1
Neuroendocrine carcinoma	5	1.5	5	0
Metaplastic carcinoma	1	0.3	1	0
Microinvasive carcinoma	10	3	10	0
T stage				
1	206	61.5	191	15
2	126	37.6	113	13
3	3	0.9	3	0
4	0	0	0	0
N stage				
0	180	53.7	173	7
1	98	29.3	90	8
2	41	12.2	38	3
3	16	4.8	6	10
LVI status				
−	220	65.7	205	15
+	115	34.3	102	13
TNM stage				
I	133	39.7	127	6
II	142	42.4	133	9
III	60	17.9	47	13
Percentage of ER expression (%)				
Mean (SD)	79.36 (23.53)		80.33 (22.42)	68.62 (32.05)
Percentage of PR expression (%)				
Mean (SD)	56.69 (34.57)		56.28 (33.96)	39.43 (37.12)
< 20	72	21.5	60	12
≥ 20	263	78.5	247	16
Ki67 status (%)				
Mean (SD)	25.12 (22.02)		23.93 (21.14)	38.21 (27.19)
< 60	292	87.2	274	18
≥ 60	43	12.8	33	10
P53 status				
−	176	52.5	163	13
+	159	47.5	144	15

### Prognostic factors

First, the expression of Ki67, ER, and PR in cancerous tissue areas was quantified as 1–100, which was the percentage of tumor nuclei positive (positive nuclei) over all the tumor nuclei (positive nuclei and negative nuclei) by manual counting. The prognostic factors included in this study were divided into categorical or continuous factors. Table 2 lists the prognostic factors included in this study. Second, to determine the cutoff values for these continuous variables (Ki67, ER, PR, and age), we transformed each continuous factor (Ki67, ER, PR, and age) into a series of binary variables using each observed value as a partition point. Table 3 provides the possible partition points for the four continuous factors. Each partition point converted the continuous variable into one binary variable. Taking Ki67 as an example, point 60% converted Ki67 into one binary variable named “Ki67 status (60%),” which equals 1 if the immunochemical status of Ki67 was no less than 60% and equals 0 otherwise. Additionally, it was mandatory that each sample size of each binary variable was not less than 10. For each patient case, 95 variables were determined, including categorical factors (histological grade, histological type, stage, T, N, P53, and LVI), continuous variables (Ki67, ER, PR, and age), and a series of binary variables. Based on a previous clinic study and clinical practice [23, 24], we introduced four potential interaction terms: T and N (*n* = 1), ER and PR (*n* = 16 × 16), age and ER (*n* = 41 × 16), and age and PR (*n* = 41 × 16). Therefore, we finally determined 1664 variables with the addition of interaction terms. The 1664 variables are the above 95 variables and four potential interaction terms: T and N (*n* = 1), ER and PR (*n* = 16 × 16), age and ER (*n* = 41 × 16), and age and PR (*n* = 41 × 16).

**Table 2:** Original prognostic factors included in this study

Prognostic factor	Original prognostic factors	*n*
Categorical factors	Histological grade, histological type, stage, T, N, LVI, P53	7
Continuous factors	Ki67, ER, PR, age	4

**Table 3:** Partition points of the continuous factors Ki67, ER, PR, and age

Prognostic factor	Partition points	*n*
Ki67 (%)	1, 5, 10, 15, 20, 25, 30, 35, 40, 45, 50, 60, 70, 80	14
ER (%)	10, 15, 20, 30, 40, 50, 60, 65, 70, 75, 80, 85, 90, 95, 98	15
PR (%)	5, 10, 15, 20, 25, 30, 40, 50, 60, 65, 70, 80, 85, 90, 95	15
Age (years)	37, 38, 39, … … , to 75, 77	40

### Analysis of patient survival times

**Fig. 2:**
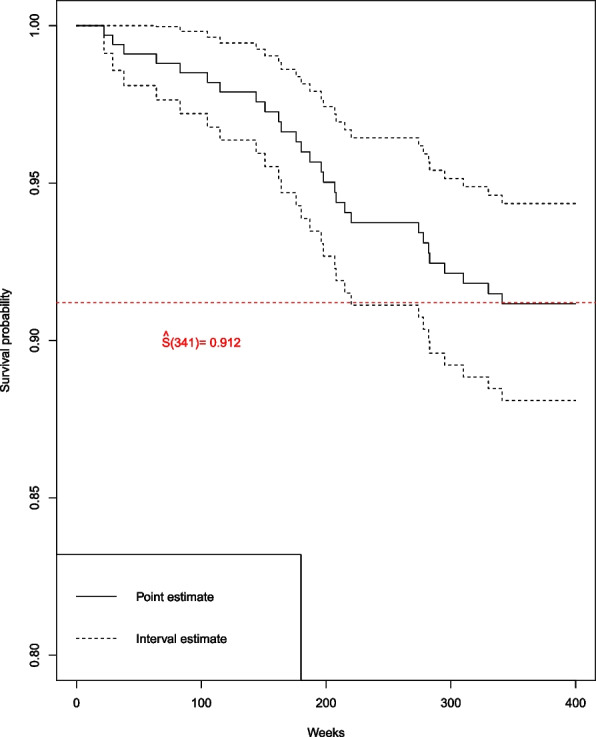
Kaplan-Meier survival probability curves

On September 15, 2016, 311 of the 335 patients were still being followed, and 24 had been lost to follow-up (including one noncancer death). Among the 311 patients, 28 patients had died from breast cancer. The clinicopathologic characteristics of the 28 patients who died from breast cancer are presented in Additional file 1: Appendix 1. The median follow-up duration was 370 weeks, with a censoring rate of 91.6%. The longest survival time of patients who died was 341 weeks. The survival probability at 341 weeks (approximately 6 and a half years) of breast cancer patients was 91.2% (95% confidence interval 88.1–94.3%) (Fig. 2).

### Prognostic model development

To determine the cutoff values for continuous variables (Ki67, ER, PR, and age), we transformed each continuous factor (Ki67, ER, PR, and age) into a series of binary variables using each observed value as a partition point. We calculated the hazard ratios and their 95% confidence limits for these binary variables (see Additional file 2:Appendix 2). It was meaningful to look for significant cutoff values for these four factors during modeling; we achieved this objective and focused on the specific prognostic models below.

#### Cox proportional hazards model without interaction terms

We first introduced 95 variables without interaction items into the model, and a SCAD variable selection method [19] was used to develop the Cox proportional hazard model (named model 1).

1 $$\mathrm{h}(t)={h}_0(t)\exp \left\{1.38{x}_1+1.01{x}_2+1.643{x}_3-0.949{x}_4+1.04{x}_5\right\}$$

The prognostic factors, beta coefficients, hazard ratios for prognostic factors, and *P*-values of *Z*-tests are provided in Table 4.

**Table 4:** Prognostic factors, coefficients, hazard ratios, and *P*-values of the *Z*-tests in model 1

Variable	Prognostic factor	Coefficient	*HR* (95% *CI*)	*p*-value
x_1_	Histological grade (I, II, III)	1.380	3.976 (1.851, 8.843)	< 0.001
x_2_	N status (0, 1, 2, 3)	1.010	2.746 (1.959, 3.849)	< 0.001
x_3_	Ki67 status (60%)	1.643	5.172 (2.288, 11.691)	< 0.001
x_4_	PR status (20%)	−0.949	0.387 (0.181, 0.826)	0.014
x_5_	Age (55 years)	1.040	2.828 (1.285, 6.222)	0.01

*HR* hazard ratio, *CI* confidence interval. Ki67 status (60%), a binary variable that is one if Ki67 is no less than 60% and zero otherwise. PR status (20%), a binary variable that is one if PR is no less than 20% and zero otherwise. Age (55 years), a binary variable that is one if age is no less than 55 years and zero otherwise

The multivariable Cox regression analysis for BCSM without interaction terms, model 1, showed that histological grade, N status, Ki67 status, PR status, and age were statistically significant predictors of patient survival. The cutoff value for Ki67 was 60%, the cutoff value for PR was 20%, and the cutoff value for age was 55 years old.

#### Cox proportional hazards model with interaction terms

Second, 1664 variables including interaction terms were used in the model to evaluate the interaction effect between prognostic factors. A SCAD variable selection method [19] was also used to develop the Cox proportional hazard model (named Model 2).

2 $$\mathrm{h}(t)={h}_0(t)\exp \left\{1.392{x}_1+0.995{x}_2+1.595{x}_3+1.202{x}_5-1.157{x}_6\right\}$$

The prognostic factors, beta-coefficients, hazard ratios for prognostic factors, and *P*-values of *Z*-tests are provided in Table 5.

**Table 5:** Prognostic factors, coefficients, hazard ratios, and *P*-values of the *Z*-tests in model 2

Variable	Prognostic factor	Coefficient	*HR* (95% *CI*)	*p*-value
*x*_1_	Histological grade (I, II, III)	1.392	4.024 (1.865, 8.684)	< 0.001
*x*_2_	*N* status (0, 1, 2, 3)	0.995	2.706 (1.929, 3.795)	< 0.001
*x*_3_	Ki67 status (60%)	1.595	4.930 (2.181, 11.143)	< 0.001
*x*_5_	Age (55 years)	1.202	3.325 (1.496, 7.389)	0.003
*x*_6_	PR − age (Age ≥ 41 years, PR ≥ 20%)	−1.157	0.315 (0.148, 0.669)	0.003

*HR* hazard ratio, *CI* confidence interval. Ki67 status (60%), a binary variable that is one if Ki67 is no less than 60% and zero otherwise. Age (55 years), a binary variable that is one if age is no less than 55 years and zero otherwise. PR − age (age ≥ 41 years, PR ≥ 20%), a binary variable, an interaction item between age and PR, which is one if age is no less than 41 years and PR is no less than 20% and zero otherwise

Model 2 showed that there might be an interaction effect between PR (20%) and age (41 years). However, PR (20%) and age (41 years) were not statistically significant predictors of patient survival in Model 2. These results indicated that Model 2 may not be self-sufficient.

#### Extended Cox prognostic model

To further optimize the two above Cox models, we first included all variables in model 1 and model 2, which were histological grade, N status, Ki67 status (60%), PR status (20%), age (55 years), age (41 years), and PR-age (age ≥ 41years, *PR* ≥ 20%), in the Cox model. The outcome indicated that PR status (20%), age (41 years old), and PR-age were not statistically significant. Furthermore, taking previous clinical experience into account, we were aware that the interaction between PR and age might vary according to the different time periods after surgery, and that the model could be divided into two parts by a specific time point. To determine the optimal time point, we considered both death and censored time periods. We developed a new Cox model 3 (named the “extended Cox prognostic model”) with a time threshold of 164 weeks after surgery, based on the smallest AIC value.

3 $$\mathrm{h}\left(\mathrm{t}\right)=\left\{\begin{array}{c}{h}_0(t)\exp \left\{A\right\},\kern10.5em t\le 164 weeks\\{}{h}_0(t)\exp \left\{A+1.594{x}_8\right\},\kern5.25em t>164 weeks\end{array}\right.$$

$$\mathrm{A}=1.329{x}_1+0.972{x}_2+1.65{x}_3-1.802{x}_4+1.378{x}_5-1.563{\mathrm{x}}_7$$

The prognostic factors, beta-coefficients, hazard ratios for prognostic factors, and *P*-values of *Z*-tests are provided in Tables 6 and 7.

**Table 6:** Prognostic factors, coefficients, hazard ratios, and *P*-values of the *Z*-tests in the extended Cox model

Variable	Prognostic factor	Coefficient	*HR* (95% *CI*)	*p*-value
x_1_	Histological grade (I, II, III)	1.329	3.777 (1.739, 8.204)	< 0.001
x_2_	*N* status (0, 1, 2, 3)	0.972	2.644 (1.872, 3.734)	< 0.001
x_3_	Ki67 status (60%)	1.650	5.208 (2.311, 11.738)	< 0.001
x_4_	PR status (20%)	−1.802	0.165 (0.050, 0.548)	0.003
x_5_	Age (55 years)	1.378	3.968 (1.548, 10.171)	0.004
x_7_	Age (41 years)	−1.563	0.210 (0.053, 0.829)	0.026
x_8_	PR − age after 164 weeks (Age ≥ 41 years, PR ≥ 20%)	1.594	4.922 (1.039, 23.325)	0.045

*HR*, hazard ratio, *CI*, confidence interval. Ki67 status (60%), a binary variable that is one if Ki67 is no less than 60% and zero otherwise. PR status (20%), a binary variable that is one if PR is no less than 20% and zero otherwise. Age (55 years), a binary variable that is one if age is no less than 55 years and zero otherwise. Age (41 years), a binary variable that is one if age is no less than 41 years and zero otherwise. PR-age after 164 weeks (age ≥ 41 years, *PR* ≥ 20%), a binary variable, an interaction item between age and PR status after 164 weeks after surgery, which is one if age is no less than 41 years old and PR is no less than 20% and zero otherwise

**Table 7:** Coefficients, hazard ratios (95% CI), and *P*-values from the prognostic model

Prognostic factor	Coefficient	*HR*	95% *CI*	*p*-value
Histological grade				
I	0	1		
II	1.33	3.78	1.74 to 8.20	< 0.001
III	2.66	14.29	3.03 to 67.24	< 0.001
N status				
0	0	1		
1	0.97	2.64	1.87 to 3.73	< 0.001
2	1.94	6.96	3.50 to 13.91	< 0.001
3	2.91	18.36	6.54 to 51.90	< 0.001
Ki67 status^a^				
Low	0.00	1.00		
High	1.65	5.21	2.31 to 11.74	< 0.001
PR status^b^				
Low	0.00	1.00		
High	−1.80	0.17	0.05 to 0.55	0.003
Age^c^				
Young	0.00	1.00		
Middle aged	−1.56	0.21	0.05 to 0.83	0.025
Elderly	−0.18	0.83	0.25 to 2.72	0.761
PR-age after 164 weeks^d^				
0	0.00	1.00		
1	1.59	4.92	1.04 to 23.33	0.045

*HR* hazard ratio, *CI* confidence interval

^a^Regarding Ki67 status, the patients were divided into two groups: low (Ki67 < 60%) and high (Ki67 ≥ 60%)

^b^Regarding PR status, the patients were divided into two groups: low (PR < 20%) and high (PR ≥ 20%)

^c^Regarding age, the patients were divided into three groups: young (< 41 years old), middle aged (41–55 years old), and elderly (55 and older)

^d^PR-age after 164 weeks (age ≥ 41 years, PR ≥ 20%), a binary variable, an interaction item between age and PR status after 164 weeks after surgery, which is one if age is no less than 41 years old and PR is no less than 20% and zero otherwise

The extended Cox prognostic model determined the cutoff values for multiple continuous prognostic factors and the interaction effect between the factors. First, the cutoff values of the prognostic factors were determined by Cox prognostic model with the SCAD variable selection method [19]. Among 1664 predictors, histological grade and N status were considered categorical factors. However, the model showed that Ki67, PR, and age were also categorical factors, and the prognostic model automatically determined their reasonable cutoff values. For Ki67 expression, a cutoff value of 60% was selected to distinguish between low expression (< 60%) and high expression (≥ 60%). For PR expression, a cutoff value of 20% was selected to distinguish between low expression (< 20%) and high expression (≥ 20%). Regarding age, we categorized patients into three groups based on age at the time of surgery: the older group, who were 55 years or older; the younger group, who were less than 41 years old; and the middle-aged group, who were between 41 and 55 years old. Second, for the interaction effect, after 164-week postoperation, there was an interaction between age and PR: the older the patients with ER/PR+, HER2−, and *PR* ≥ 20% were, the lower the survival and more likely to recur and metastasize exceeding 164 weeks (more than 3 years) after surgery.

In our study, we found that the hazard ratio for BCSM of the patients with the histological grade 2 was 3.78 times as much as that of the patients with the histological grade 1. The hazard ratio for BCSM of the patients with the histological grade 3 was 14.29 times as much as that of the patients with the histological grade 1. We found that the hazard ratio for BCSM of the patients with the N1 status was 2.64 times as much as that of the patients with the N0 status. The hazard ratio for BCSM of the patients with the N2 status was 6.96 times as much as that of the patients with the N0 status. The hazard ratio for BCSM of the patients with the N3 status was 18.36 times as much as that of the patients with the N0 status. The patients with high Ki67 expression had a hazard ratio for BCSM that was 4.21 times higher than that of the patients with low Ki67 expression (see Table 7). Within 164 weeks postoperation, the hazard ratio for BCSM of the patients with high PR expression was 0.17 times as much as that of the patients with low PR expression. Within 164-week postoperation, patients aged < 41 had the highest hazard ratio for BCSM, followed by patients aged ≥ 55, while patients aged 41 to 55 showed the lowest hazard ratio for BCSM. After 164-week postoperation, there was an interaction effect between age and PR for patients aged ≥ 41 years and *PR* ≥ 20%. The hazard ratio for BCSM in patients aged ≥ 41 years and *PR* ≥ 20% was elevated after 164-week postoperation. For patients with high PR expression, age was positively correlated with mortality after 164-week postoperation. For patients with high PR expression after 164-week postoperation, the patients aged 41 to 55 had nearly the same hazard ratio for BCSM as those aged < 41, while the hazard ratio for BCSM of patients aged ≥ 55 years was 3.09 times higher than that of patients aged < 41 years (see Table 8).

**Table 8:** An interaction effect between age and PR for patients aged ≥ 41 years and *PR* ≥ 20% after 164-week postoperation

The different time periods	Prognostic factor				Coefficient	*HR*
< 164-week postoperation	PR status	Low			0.00	1.00
		High			−1.80	0.17
	Age^a^	Young			0.00	1.00
		Middle aged			−1.56	0.21
		Elderly			−0.19	0.83
≥ 164-week postoperation	PR status	Low	Age^a^	Young	0.00	1.00
				Middle aged	−1.56	0.21
				Elderly	−0.19	0.83
		High	Age^a^	Young	−1.80	0.17
				Middle aged	−1.77	0.17
				Elderly	−0.39	0.68

*HR* hazard ratio. ^a^Regarding age, the patients were divided into three groups: young (< 41 years old), middle aged (41–55 years old), and elderly (55 and older)

### Utilizing the nomogram

**Fig. 3:**
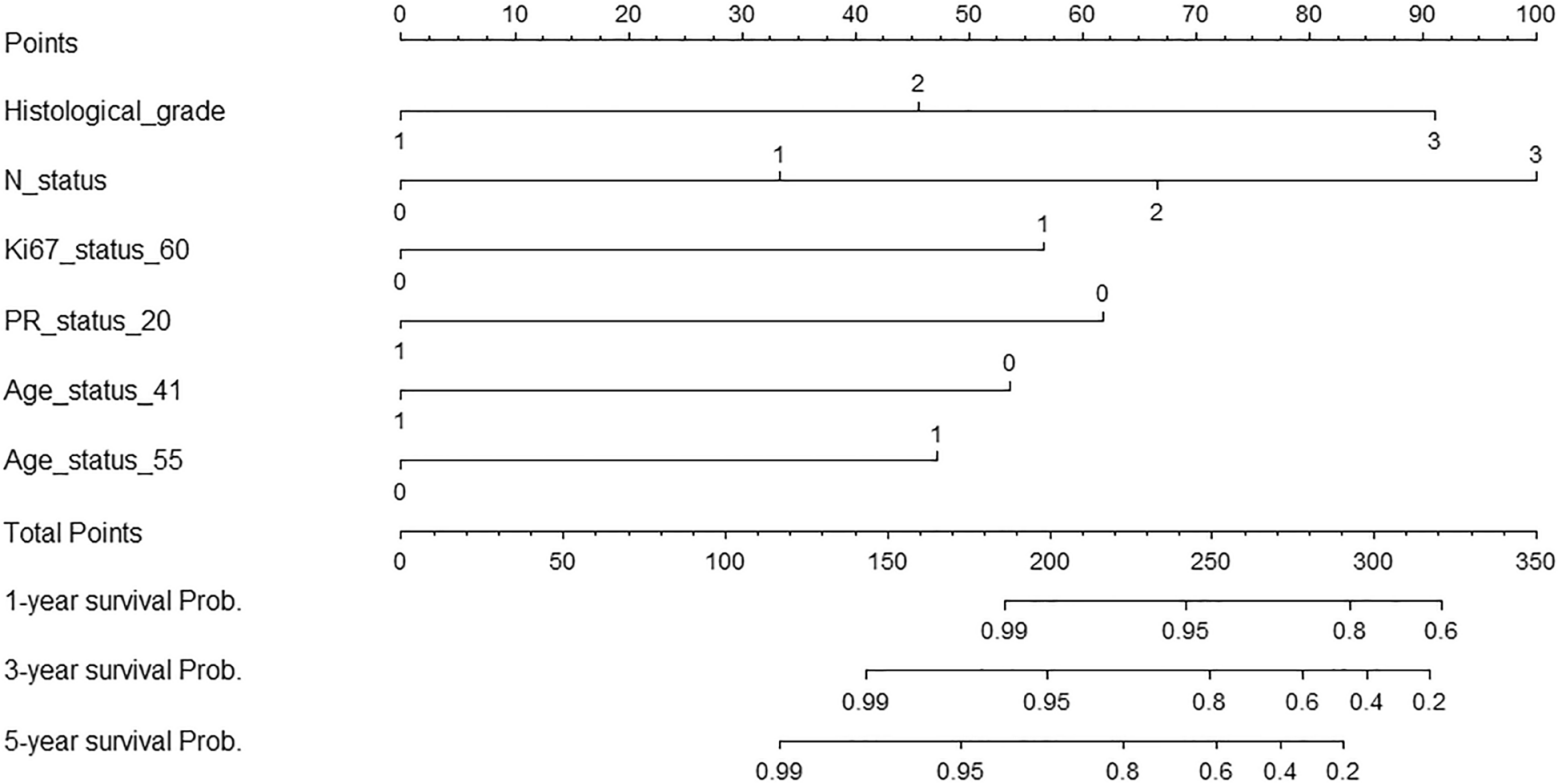
Nomogram of 1-year, 3-year, and 5-year survival probability

To improve the practicability of our model, we established a nomogram of 1-year, 3-year, and 5-year survival probability based on our model (Fig. 3). The nomogram consisted of eleven rows. The first “points” row was the point assignment for each variable. For an individual patient, each variable (rows 2–7) was assigned a point value by drawing a vertical line between the exact variable value and the first “points” row. Subsequently, the total points could be obtained by summing all of the allotted points for the six variables (rows 2–7). The total point sum was found in the “total points” row. Finally, the 1-, 3-, and 5-year survival probability could be predicted by drawing a vertical line between the “total points” row and the “predicted probability” rows (rows 9–11, respectively).

### Model discrimination and calibration

**Fig. 4:**
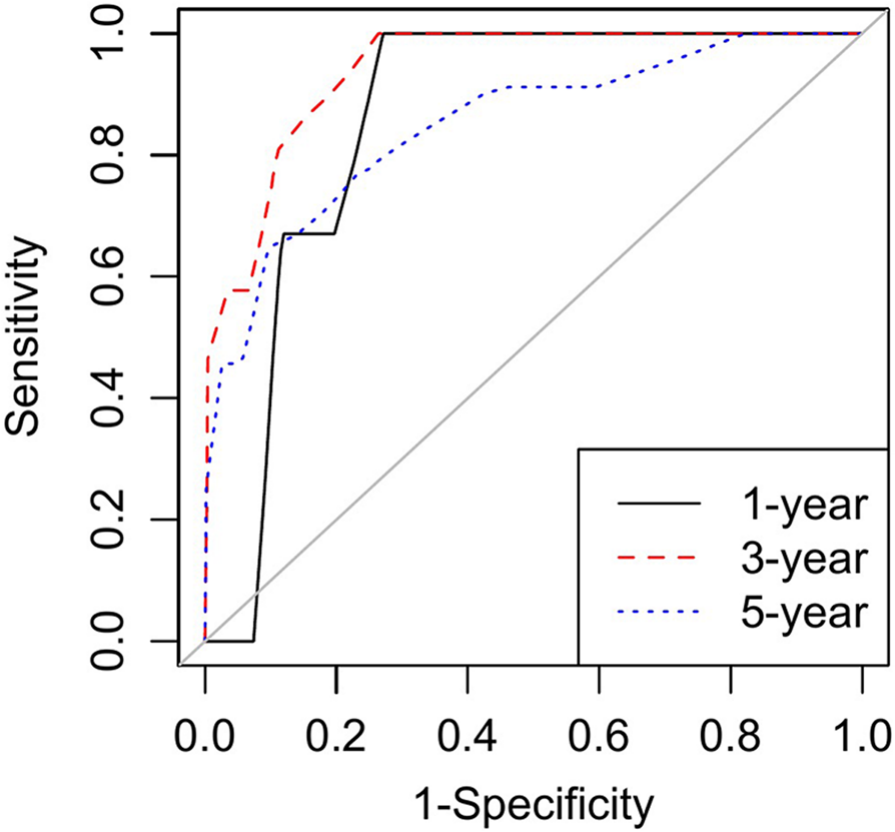
ROC curves for our extended Cox prognostic model at 1-year, 3-year, and 5-year postoperation

As expected, this extended Cox prognostic model showed good discrimination. Figure 4 shows the ROC curves for BCSM at 1 year, 3 years, and 5 years postoperatively. The AUCs for BCSM at 1 year, 3 years, and 5 years postoperatively are shown in Table 9. The AUC values for BCSM at 1 year, 3 years and 5 years postoperatively were all larger than 0.80. The AUC value for BCSM at 3-year postoperation was as high as 0.94, with a 95% CI from 0.89 to 0.99. Therefore, our extended Cox prognostic model showed good discrimination.

**Table 9:** The AUC and 95% CI for our extended Cox prognostic model at 1-year, 3-year, and 5-year postoperation

	AUC	95% *CI*
1 year	0.85	0.78 to 0.93
3 years	0.94	0.89 to 0.99
5 years	0.81	0.68 to 0.94

*CI* Confidence interval

The extended Cox prognostic model was also well calibrated by the GOF test [21]. We grouped the risk scores into 10 groups and then calculated the GOF statistic 7.37, with a *P*-value of 0.6 (> 0.05) [22]. These results indicated that our extended Cox prognostic model fit was good.

## Discussion

In this study, we explored an extended Cox model for the prognosis of ER/PR+ and HER2− breast cancer, with calculating the cutoff points of prognostic factors and their interaction. The cutoff values of Ki67, PR, and age and the interaction between age and PR status were generated from model calculations. The model was well calibrated and provided a high degree of discrimination.

Here, we found that the prognosis of patients was associated with histological grade, N stage, Ki67 and PR statuses, and age. We found that the cutoff values for PR, Ki67, and age were 20%, 60%, and 41–55 years, respectively. It is important to point out that the cutoff values of prognostic factors, including Ki67, PR, and age, were determined only based on our extended Cox prognostic model (a multivariable analysis) and not based on univariate analysis, experience, or speculation; this is different from previous studies [13–15, 25, 26]. The prognosis of patients was associated with histological grade, N stage, Ki67 and PR statuses, and age, which was consistent with previous reports [23, 27]. Histological grade and N stage had a linear effect on the hazard ratio. The cutoff value (20%) for PR was consistent with other studies [28, 29], which indicated the high fidelity of our model. We believe that breast cancer with low PR expression is probably a different intrinsic subtype of breast cancer, that is, the luminal B subtype. Our prognostic model determined 60% as the cutoff value for Ki67 status, which was much higher than in other studies (e.g., 14%, 20%, 25%, and 30%) [23, 28–31]. We speculated that this situation was because of a lack of a standardized procedure for Ki67 assessment and the controversial Ki67 assay interpretation [30, 31] and could also be attributed to their determining the cutoff value for Ki67 with the ROC method (a univariate analysis) used in a previous study [25, 26] rather than the multivariate analysis method that we adopted. Therefore, the prognostic value of the Ki67 index in breast cancer needs to be further explored. The cutoff value for age (41 and 55 years) was consistent with the age range of perimenopausal and menopausal Chinese women, although it was different from those of other studies [8, 13, 15, 23, 27]. Hence, our models showed high consistency to the currently available gold standard, such as the PR factor. The values are well correlated with the physiological conditions of our patients, such as age. Most importantly, our model generates the cutoff values based on algorithm calculation without empirical biases or univariate analysis.

In our study, algorithmic analysis with the extended Cox prognostic model showed that there was an interaction between age and PR for patients aged ≥ 41 years and *PR* ≥ 20% after 164-week postoperation. The interaction between age and PR caused the patients with age ≥ 41years and *PR* ≥ 20% to have relatively higher mortality after 164-week postoperation. The existence of the interaction between age and PR status has been previously reported [23, 24]. We feel that our study of the interaction was deeper and clearer. We believe that the existence of the interaction may be related to multiple factors, including the subtype of breast cancer, the status of menopause, and the health of the patients 164 weeks after surgery. However, the specific causes remain to be further studied. Meanwhile, we found that the older the patients with ER/PR+, HER2−, and *PR* ≥ 20% were, the lower the survival was and the more likely recurrence and metastasis were 164 weeks after surgery. Therefore, the clinicians should pay more attention to the long-term follow-up of ER/PR+, HER2−, and *PR* ≥ 20% subtype breast cancer patients aged ≥ 41years (equivalent to the peri- and postmenopausal period in China) more than 3 years after surgery. Drug selection in adjuvant endocrine therapy requires careful consideration in these patients. Endocrine therapy should be sufficiently extended for these patients.

Compared with those previous studies, our studies showed the following four advantages: (1) we adopted a new concept to determine the cutoff values based only on our extended Cox prognostic model, a multivariable analysis. The cutoff values for multiple prognostic factors, including age/PR/Ki67 status, were determined and were statistically significant for prognosis. (2) During the development of the model, we introduced potential interactions and surmised that the model could be divided into two parts by a specific time point. An extended Cox model with a time threshold of 164-week postoperation was built based on statistical analysis. We found that there was an interaction between age and PR at 164-week postoperation. The hazard ratio for BCSM in patients aged ≥ 41years and *PR* ≥ 20% was elevated after 164-week postoperation. Therefore, clinicians should pay more attention to the long-term follow-up of ER/PR+, HER2−, *PR* ≥ 20% subtype breast cancer patients aged ≥ 41years more than 3 years after surgery. (3) In actual clinical practice, we developed a user-friendly nomogram based on our extended Cox model to facilitate clinical application. The prediction factors in our model were all included in domestic routine testing covered by Chinese healthcare insurance with standardized test norms. There was no additional financial cost. Clinicians could predict the 1-, 3-, and 5-year survival probability in a specific patient by summing the scores of each variable based on our nomogram. (4) Our study was derived from Chinese clinical data, which could be the most relevant model for Chinese clinical practice. Due to gene similarity, the model could also apply to the prognosis of breast cancer in other Asian female populations. The cutoff value for age (41 and 55 years) was consistent with the age range of perimenopausal and menopausal Chinese women, although it was different from those of other studies.

One limitation of our study was that it was validated internally, but it lacked external validation. It would be better to perform external validation to validate the model for overfitting. Another limitation was that the overall sample size and the number of outcome events were relatively small. This might cause possible bias in the results. In addition, this was a single-center retrospective study and was thus vulnerable to selection bias. Therefore, whether our model and our cutoff values can be generalized to other populations needs further investigation.

In conclusion, using the new modeling concept and statistical method, an extended Cox prognostic model for the prognosis of ER/PR+ and HER2− breast cancer was explored while determining the cutoff points of prognostic factors and their interaction. The results of our study offer guidance for the prognosis and treatment of patients with ER/PR+ and HER2− breast cancer in China. Moreover, we adopted a new concept to determine the cutoff values of the continuous factors, introduced potential interactions, and surmised that the model could be divided into two parts by a specific time point. The new conceptualization and statistical method in our study were different from those in previous studies. The new modeling concept used in our study will likely become a research method for prognostic cutoff values. This study has significance as a reference for the development of similar study in the future.

## Supplementary Information

**Additional file 1: Appendix 1.** Clinicopathologic characteristics of 28 deaths.

**Additional file 2: Appendix 2.** Hazard ratios and their 95% confidence limits of Ki67, PR, ER, and age. **Figure 1.** Hazard ratios and their 95% confidence limits for a series of binary variables of Ki67. **Figure 2.** Hazard ratios and their 95% confidence limits for a series of binary variables of ER. **Figure 3.** Hazard ratios and their 95% confidence limits for a series of binary variables of PR. **Figure 4.** Hazard ratios and their 95% confidence limits for a series of binary variables of age.

## Data Availability

All data generated or analyzed during this study are included in this published article.

## Abbreviations

ER: Estrogen receptor
PR: Progesterone receptor
HER2: Human epidermal growth factor receptor 2
LVI: Lymphovascular invasion
BCSM: Breast cancer-specific mortality
SCAD: Smoothly clipped absolute deviation
