# Supplementary material for: Development and validation of an extended Cox prognostic model for patients with ER/PR+ and HER2− breast cancer: a retrospective cohort study
Source: World J Surg Oncol. 2022 Oct 12;20:338. doi: 10.1186/s12957-022-02790-0 (PMC9555115; doi:10.1186/s12957-022-02790-0)
Supplement: Supplementary file 1 — Additional file 1: Appendix 1. Clinicopathologic characteristics of 28 deaths. [file 12957_2022_2790_MOESM1_ESM.docx]

Appendix 1. Clinicopathologic characteristics of 28 deaths

| Age | Date of surgery | Date of death | Pathological Type | TNM stage | Histologic Grade | T stage | N stage | LVI status | Percentage of ER expression (%) | Percentage of PR expression (%) | P53 status | Ki67 status (%) |
| --- | --- | --- | --- | --- | --- | --- | --- | --- | --- | --- | --- | --- |
| 58 | 2009/2/6 | 2015/6/11 | invasive carcinoma of no special type | 2 | 2 | 1 | 1 | 0 | 90 | 10 | 1 | 10 |
| 60 | 2009/4/3 | 2014/8/3 | invasive carcinoma of no special type | 3 | 2 | 1 | 3 | 1 | 95 | 40 | 0 | 10 |
| 56 | 2009/4/13 | 2014/7/18 | invasive carcinoma of no special type | 1 | 3 | 1 | 0 | 1 | 98 | 90 | 1 | 60 |
| 59 | 2009/4/23 | 2014/9/18 | invasive carcinoma of no special type | 2 | 2 | 2 | 1 | 0 | 90 | 30 | 1 | 30 |
| 39 | 2009/4/30 | 2013/7/18 | invasive carcinoma of no special type | 3 | 3 | 2 | 2 | 0 | 50 | 70 | 1 | 50 |
| 67 | 2009/5/5 | 2015/4/14 | invasive carcinoma of no special type | 2 | 3 | 2 | 1 | 0 | 90 | 80 | 0 | 10 |
| 56 | 2009/5/8 | 2015/1/2 | invasive carcinoma of no special type | 2 | 2 | 1 | 1 | 0 | 90 | 15 | 0 | 0 |
| 48 | 2009/6/3 | 2010/2/25 | invasive carcinoma of no special type | 3 | 2 | 1 | 2 | 0 | 20 | 0 | 0 | 0 |
| 54 | 2009/6/12 | 2015/12/25 | micropapillary carcinomas | 2 | **2** | 1 | 1 | 1 | 90 | 60 | 1 | 25 |
| 56 | 2009/6/24 | 2012/5/20 | invasive carcinoma of no special type | 2 | 3 | 2 | 1 | 1 | 95 | 5 | 0 | 80 |
| 59 | 2009/7/13 | 2011/2/15 | invasive carcinoma of no special type | 3 | 2 | 2 | 3 | 0 | 5 | 0 | 1 | 40 |
| 34 | 2009/7/15 | 2011/7/20 | invasive carcinoma of no special type | 3 | 3 | 2 | 3 | 1 | 70 | 30 | 1 | 70 |
| 80 | 2009/7/30 | 2010/10/23 | invasive carcinoma of no special type | 3 | 3 | 1 | 3 | 0 | 70 | 60 | 1 | 70 |
| 47 | 2009/8/5 | 2013/9/18 | invasive carcinoma of no special type | 3 | 3 | 2 | 2 | 1 | 70 | 60 | 0 | 10 |
| 50 | 2009/8/18 | 2012/5/22 | invasive carcinoma of no special type | 2 | 2 | 2 | 1 | 1 | 5 | 0 | 1 | 70 |
| 68 | 2009/8/20 | 2013/3/24 | invasive carcinoma of no special type | 3 | 2 | 1 | 3 | 0 | 90 | 80 | 1 | 30 |
| 55 | 2009/8/21 | 2010/1/25 | invasive carcinoma of no special type | 1 | 2 | 1 | 0 | 1 | 90 | 0 | 0 | 70 |
| 62 | 2009/8/25 | 2012/10/7 | invasive carcinoma of no special type | 1 | 3 | 1 | 0 | 0 | 10 | 5 | 0 | 60 |
| 57 | 2009/9/3 | 2013/6/24 | invasive carcinoma of no special type | 2 | 2 | 1 | 1 | 1 | 90 | 20 | 0 | 20 |
| 50 | 2009/9/17 | 2013/3/3 | invasive carcinoma of no special type | 1 | 2 | 1 | 0 | 0 | 80 | 70 | 0 | 70 |
| 69 | 2009/9/24 | 2013/9/21 | invasive lobular carcinomas | 3 | 2 | 1 | 3 | 1 | 10 | 0 | 0 | 10 |
| 47 | 2009/10/29 | 2010/5/26 | invasive carcinoma of no special type | 2 | 3 | 2 | 0 | 0 | 60 | 0 | 0 | 80 |
| 55 | 2009/11/19 | 2015/4/23 | invasive carcinoma of no special type | 3 | 2 | 2 | 3 | 1 | 95 | 95 | 1 | 70 |
| 60 | 2009/12/1 | 2012/2/20 | micropapillary carcinomas | 3 | 3 | 2 | 3 | 1 | 50 | 0 | 0 | 10 |
| 40 | 2009/12/4 | 2013/11/26 | invasive carcinoma of no special type | 1 | 2 | 1 | 0 | 0 | 95 | 90 | 1 | 25 |
| 36 | 2009/12/16 | 2013/2/9 | invasive carcinoma of no special type | 3 | 3 | 2 | 3 | 0 | 40 | 5 | 1 | 40 |
| 65 | 2009/12/25 | 2013/5/13 | invasive papillary carcinomas | 1 | 2 | 1 | 0 | 0 | 90 | 90 | 0 | 10 |
| 72 | 2009/12/30 | 2013/10/6 | micropapillary carcinomas | 3 | 3 | 2 | 3 | 1 | 99 | 99 | 0 | 40 |
