# Supplementary figures and images for: Development and validation of an extended Cox prognostic model for patients with ER/PR+ and HER2− breast cancer: a retrospective cohort study
Source: World J Surg Oncol. 2022 Oct 12;20:338. doi: 10.1186/s12957-022-02790-0 (PMC9555115; doi:10.1186/s12957-022-02790-0)

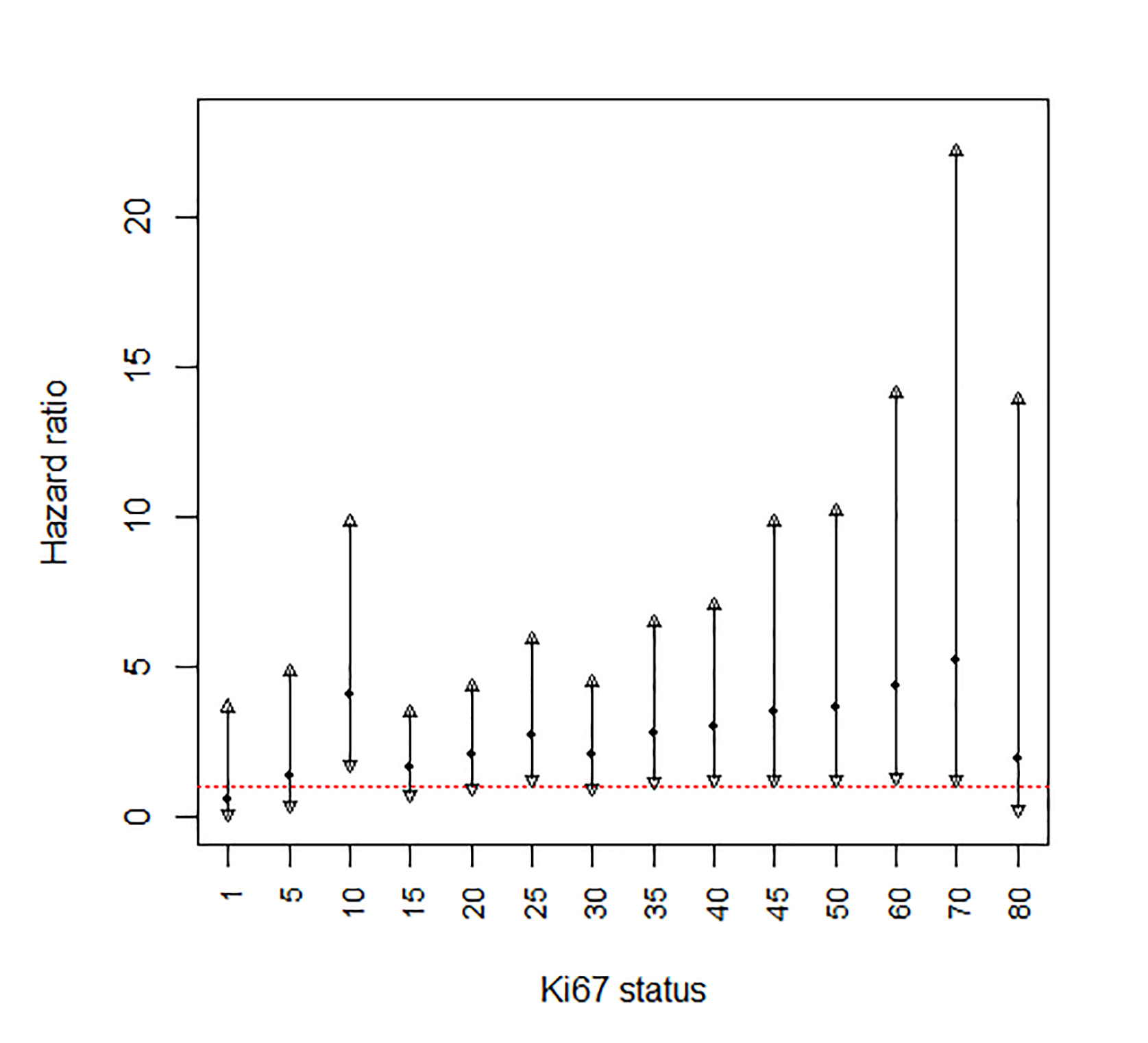

Supplement: Supplementary file 2 — Additional file 2: Appendix 2. Hazard ratios and their 95% confidence limits of Ki67, PR, ER, and age. Figure 1. Hazard ratios and their 95% confidence limits for a series of binary variables of Ki67. Figure 2. Hazard ratios and their 95% confidence limits for a series of binary variables of ER. Figure 3. Hazard ratios and their 95% confidence limits for a series of binary variables of PR. Figure 4. Hazard ratios and their 95% confidence limits for a series of binary variables of age. [file 12957_2022_2790_MOESM2_ESM.zip › Appendix 2. Figure 1. (revision).tif]

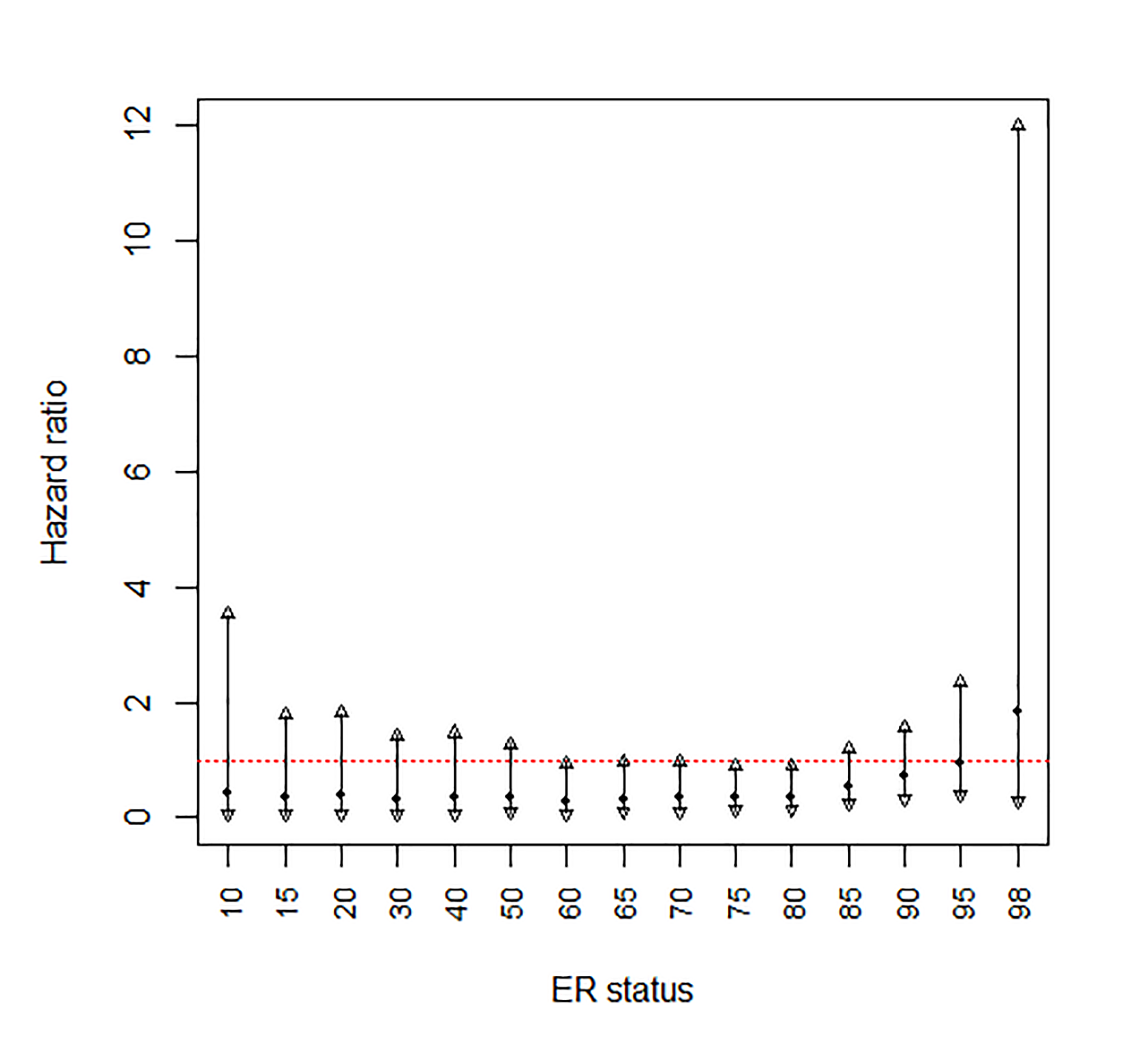

Supplement: Supplementary file 2 — Additional file 2: Appendix 2. Hazard ratios and their 95% confidence limits of Ki67, PR, ER, and age. Figure 1. Hazard ratios and their 95% confidence limits for a series of binary variables of Ki67. Figure 2. Hazard ratios and their 95% confidence limits for a series of binary variables of ER. Figure 3. Hazard ratios and their 95% confidence limits for a series of binary variables of PR. Figure 4. Hazard ratios and their 95% confidence limits for a series of binary variables of age. [file 12957_2022_2790_MOESM2_ESM.zip › Appendix 2. Figure 2. (revision).tif]

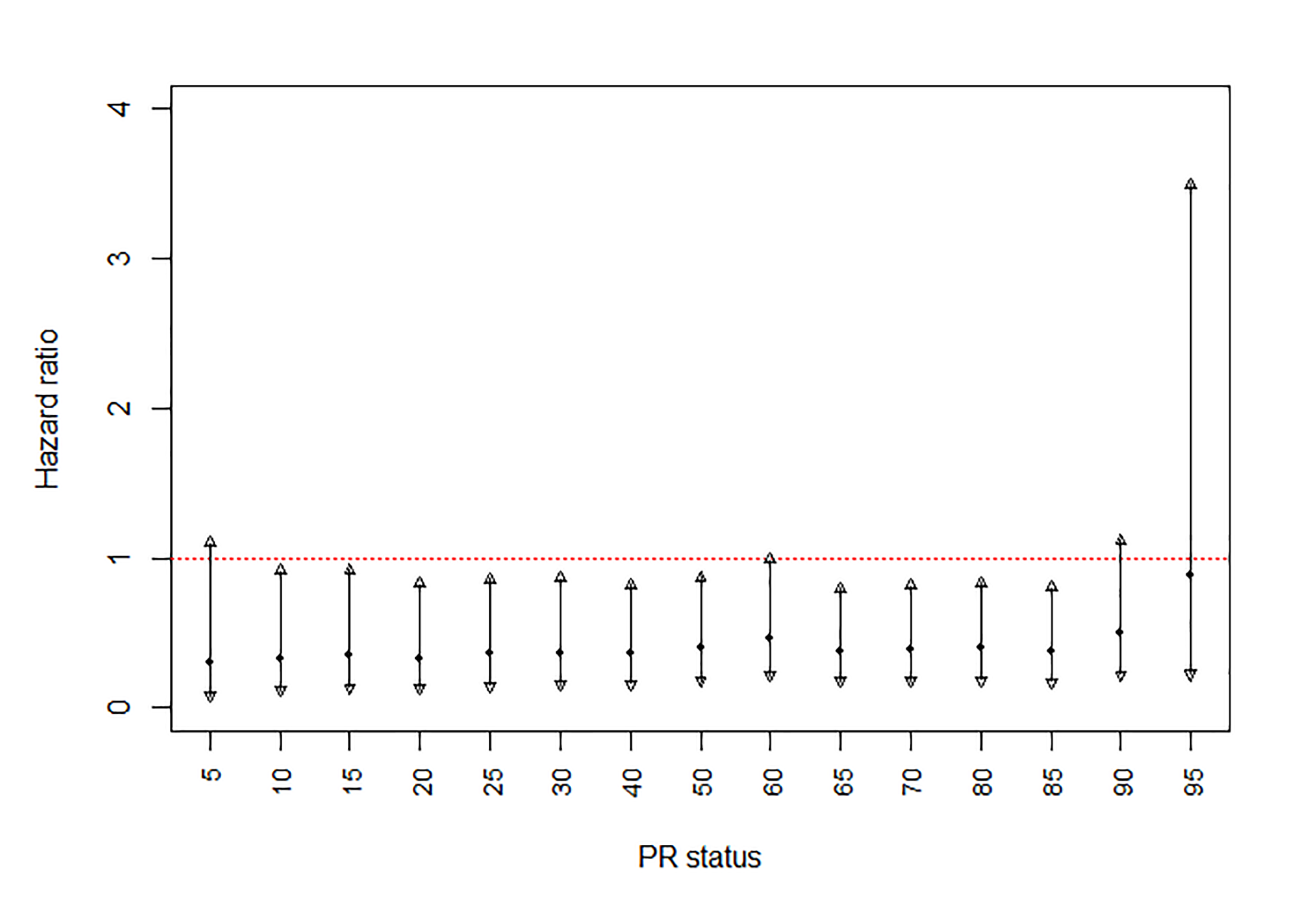

Supplement: Supplementary file 2 — Additional file 2: Appendix 2. Hazard ratios and their 95% confidence limits of Ki67, PR, ER, and age. Figure 1. Hazard ratios and their 95% confidence limits for a series of binary variables of Ki67. Figure 2. Hazard ratios and their 95% confidence limits for a series of binary variables of ER. Figure 3. Hazard ratios and their 95% confidence limits for a series of binary variables of PR. Figure 4. Hazard ratios and their 95% confidence limits for a series of binary variables of age. [file 12957_2022_2790_MOESM2_ESM.zip › Appendix 2. Figure 3. (revision).tif]

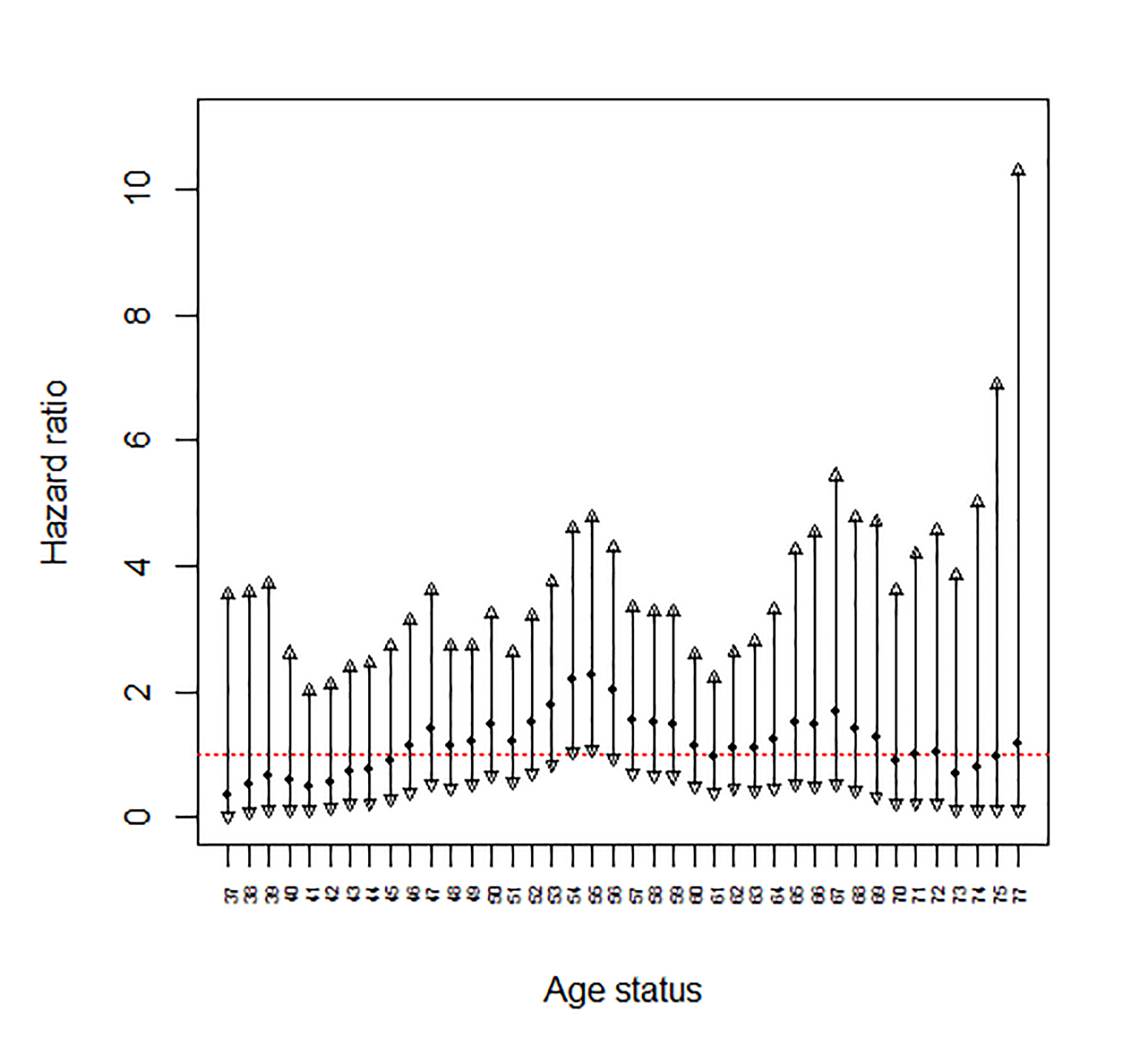

Supplement: Supplementary file 2 — Additional file 2: Appendix 2. Hazard ratios and their 95% confidence limits of Ki67, PR, ER, and age. Figure 1. Hazard ratios and their 95% confidence limits for a series of binary variables of Ki67. Figure 2. Hazard ratios and their 95% confidence limits for a series of binary variables of ER. Figure 3. Hazard ratios and their 95% confidence limits for a series of binary variables of PR. Figure 4. Hazard ratios and their 95% confidence limits for a series of binary variables of age. [file 12957_2022_2790_MOESM2_ESM.zip › Appendix 2. Figure 4. (revision).tif]
